# Risk factors for bronchiolitis obliterans syndrome after hematopoietic stem cell transplantation: a systematic review and meta-analysis

**DOI:** 10.1186/s12890-025-03925-1

**Published:** 2025-10-02

**Authors:** Guangchen Pu, Guangmin Nong, Qing Wei, Yunyan He, Wenguang Jia, Zhenhao Lu, Guosheng Qiu, Jun Xie, Xun Chen

**Affiliations:** Difficult and Critical illness Center, Pediatric Clinical Medical Research Center of Guangxi, The First Affiliated Hospital of Guangxi Medical University, Nanning, Guangxi 530021 China

**Keywords:** Hematopoietic stem cell transplantation, Bronchiolitis obliterans syndrome, Risk factor, Meta-analysis

## Abstract

**Background:**

Bronchiolitis obliterans syndrome (BOS) after hematopoietic stem cell transplantation (HSCT) is a late-onset complication that significantly impairs patients’ quality of life and is associated with a high mortality rate. Currently, the risk factors for BOS after HSCT remain controversial. We therefore conducted a systematic review and meta-analysis to identify potential risk factors associated with BOS after HSCT.

**Methods:**

Three primary medical databases (PubMed, Web of Science, Embase) were exhaustively reviewed from their inception through November 2024 to assess contributing factors for BOS occurrence following HSCT. Data synthesis was conducted using RevMan 5.4 for meta-analytic evaluation.

**Results:**

Fourteen studies involving 10,317 HSCT recipients were included, 778 of whom developed BOS. Female sex (OR = 1.26; 95% CI: 1.08, 1.47; *p* = 0.003), ABO blood group incompatibility (OR = 1.39; 95% CI: 1.07, 1.81; *p* = 0.01), peripheral blood stem cell transplantation (PBSCT) (OR = 1.31; 95% CI: 1.04, 1.64; *p* = 0.02), myeloablative conditioning (MAC) (OR = 1.63; 95% CI: 1.23, 2.16; *p* = 0.0008), acute graft-versus-host disease (aGVHD) (OR = 1.93; 95% CI: 1.16, 3.23; *p* = 0.01), grade Ⅱ-Ⅳ aGVHD (OR = 1.41; 95% CI: 1.12, 1.77; *p* = 0.004), and extrapulmonary chronic graft-versus-host disease (cGVHD) (OR = 11.69; 95% CI: 5.29, 25.82; *p* < 0.00001) were associated with an increased risk of BOS after HSCT.

**Conclusions:**

Female sex, ABO blood group incompatibility, PBSCT, MAC, aGVHD (especially grade II–IV), and extrapulmonary cGVHD are associated with an increased risk of BOS after HSCT.

**PROSPERO registration:**

CRD42024609569

**Supplementary Information:**

The online version contains supplementary material available at 10.1186/s12890-025-03925-1.

## Introduction

Hematopoietic stem cell transplantation (HSCT) has evolved into a clinically mature therapeutic approach since its inception in 1955, becoming an effective treatment for various hematologic malignancies, certain immune disorders, and specific solid tumors [1, 2]. However, there are many related complications, high incidence and substantial severity, such as bronchiolitis obliterans syndrome (BOS). Without prompt intervention, BOS carries a dismal 5-year survival rate of merely 10–13% [3]. Given its clinical gravity, the investigation of BOS risk factors has garnered substantial research attention. In recent years, research results tend to favor acute graft-versus-host disease (aGVHD), gender, unrelated donor, etc. as risk factors [4–6]. However, the conclusions regarding the risk factors for BOS after HSCT have been inconsistent, and these factors remain debatable. Therefore, this study, involving a comprehensive systematic review and meta-analysis of the existing literature, aimed to identify potential risk factors associated with the development of BOS after HSCT.

## Methods

This meta-analysis adhered to the Preferred Reporting Items for Systematic Reviews and Meta-Analyses (PRISMA) 2020 guidelines [7] (Supplementary Materials 1). The trial protocol was registered in the International Prospective Register of Systematic Reviews (PROSPERO) (CRD42024609569).

### Literature search strategy

Two independent researchers (GCP and ZHL) performed a comprehensive electronic search across PubMed, Embase, and Web of Science, covering records through November 5, 2024. The search methodology incorporated controlled vocabulary (MeSH terms) and free-text keywords related to HSCT, bronchiolitis obliterans (BO), and BOS. The complete search methodology for PubMed is available in the Supplementary Materials 2. Additionally, we manually screened reference lists of included publications.

### Inclusion and exclusion criteria

Criteria for inclusion: (1) case-control or retrospective cohort studies; (2) subjects were patients who had undergone HSCT; (3) patients meeting the 2005 or 2014 National Institutes of Health (NIH) consensus diagnostic criteria for BO or BOS [8]; (4) studies that provided complete data on BOS risk factors. Exclusion criteria included: (1) case reports or reviews; (2) BOS caused by lung transplantation or infection; (3) studies for which the full text was not available; (4) studies with unclear definitions of BO/BOS; (5) studies that did not report any BOS risk factors.

### Data extraction

Two independent researchers (GCP and JX) carried out study selection and information collection processes separately. The extracted variables encompassed: first author’s name, year of publication, nation, research type, group sample sizes, and reported risk factors. Any discrepancies were adjudicated through deliberative discussion until unanimous agreement was achieved.

### Quality assessment

Two independent researchers (QW and GSQ) conducted methodological appraisals of all included studies. The Newcastle-Ottawa Scale (NOS) was employed to evaluate observational studies, assessing three critical domains: (1) cohort selection criteria, (2) group comparability, and (3) outcome/exposure determination. This standardized tool utilizes an 8-item checklist with a maximum achievable score of 9 points. Based on total scores, studies were categorized into three quality tiers: high-quality (≥ 7 points), moderate-quality (4–6 points), and low-quality (≤ 3 points).

### Statistical analysis

Data analyses were conducted with Cochrane’s RevMan software (v5.4), computing pooled odds ratios (ORs) with 95% confidence intervals (CIs) for all potential risk factors for BOS. Statistical significance of combined effects was evaluated using two-tailed Z-tests (α = 0.05 threshold). Statistical heterogeneity among included studies was assessed using Cochran’s Q test and *I*^2^ statistics [9]. A fixed-effects model was employed for meta-analysis when *P* > 0.1 and *I*^2^ < 50%, indicating no significant heterogeneity [10]. Conversely, a random-effects model was applied when *P* ≤ 0.1 or *I*^2^ ≥ 50%, suggesting the presence of substantial heterogeneity [10]. To evaluate the robustness of findings, sensitivity analyses were performed by alternately applying fixed-effect and random-effects models to all examined risk factors, with systematic exclusion of individual studies to determine result stability. Additionally, to evaluate potential publication bias, funnel plot asymmetry analysis and Egger’s regression were employed for meta-analyses containing ten or more studies, with statistical significance set at *P* < 0.05 (two-tailed) [11, 12]. Notably, funnel plots were generated using fixed-effects models, as they demonstrate greater robustness than random-effects models in publication bias detection.

## Results

### Study selection and study characteristics

**Fig. 1:**
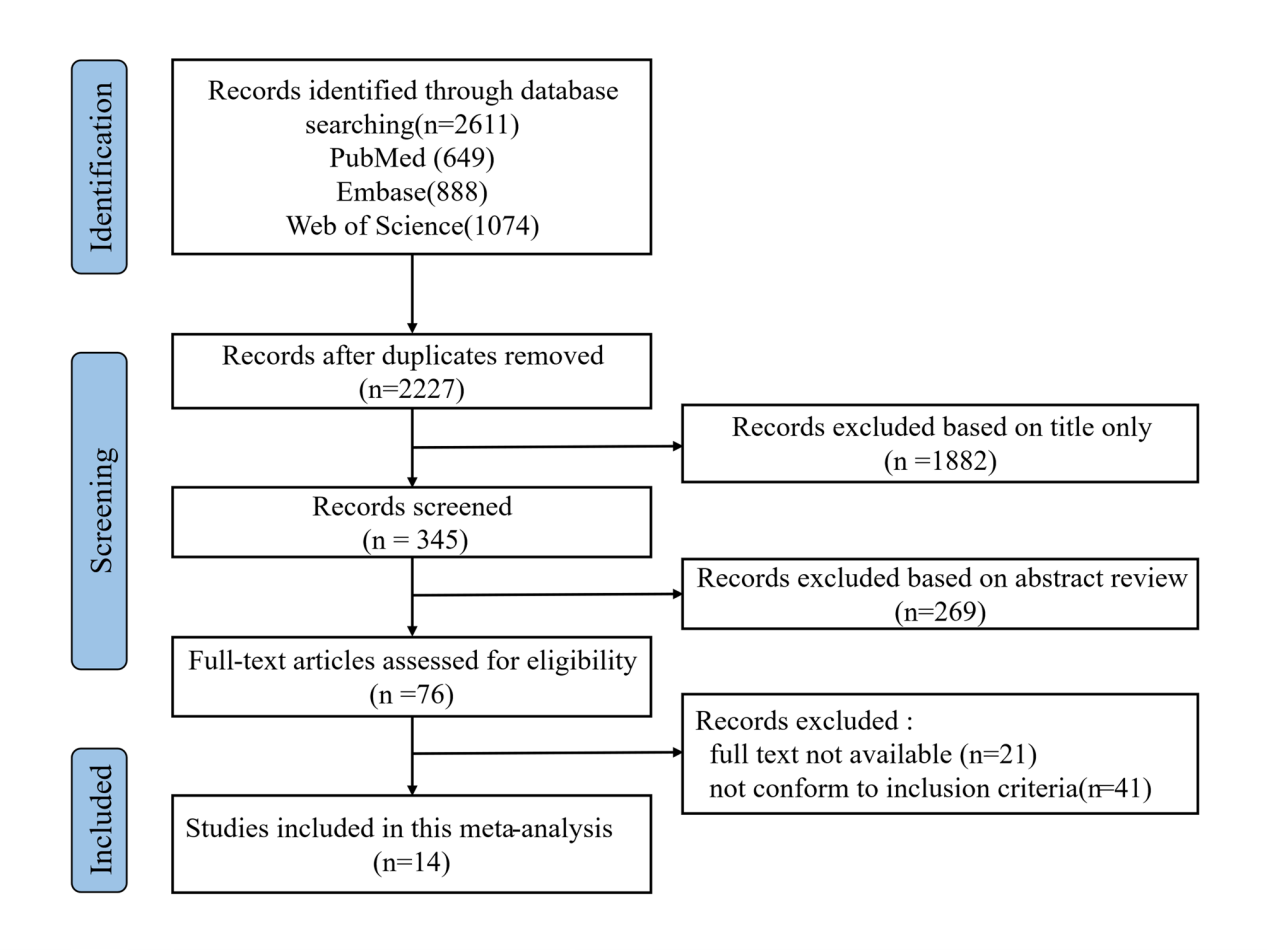
Flow chart of study selection

A total of 2,611 articles were initially identified through database searches (PubMed: *n* = 649; Embase: *n* = 888; Web of Science: *n* = 1,074). After removing 384 duplicates, 2,227 records underwent title/abstract screening. Of these, 76 studies qualified for full-text assessment, with 14 ultimately meeting the inclusion criteria for meta-analysis [13–26]. The included studies comprised 11 retrospective cohort and 3 case-control investigations, published between 2008 and 2024. Figure 1 provides a detailed overview of the systematic literature screening procedure. In total, 10,317 HSCT recipients were included in this meta-analysis, with 778 developing BOS. Table 1 summarizes the baseline characteristics and NOS scores of included studies. Complete quality assessment details are available in Supplementary Material 2 (Table S1, S2). All included studies were of high quality (NOS scores: 7–9). Finally, we estimated 13 risk factors that may influence the development of BOS in HSCT recipients.

**Table 1:** Baseline characteristics and quality assessment scores of included studies

First author	Publication year	Country	Study design	Sample size (BOS/Total)	Risk factors reported	NOS scores
Duncan CN [13]	2008	USA	Cohort	18/217	F1, F3, F4, F5, F10, F12	7
Moghadam KG [14]	2010	Iran	Cohort	19/42	F1, F5	7
Forslow U [15]	2011	USA	Cohort	25/527	F1, F4, F5, F6, F9, F11, F12	8
Au BK [16]	2011	USA	Cohort	63/945	F1, F3, F5, F6, F7, F8, F12, F13	8
Nakaseko C [17]	2011	Japan	Cohort	57/2,087	F1, F5, F6, F7, F8, F10, F11, F12	8
Mo XD [18]	2013	China	Case-control	36/144	F1, F3, F4, F7, F8, F12, F13	7
Nakasone H [19]	2013	Japan	Case-control	196/2,156	F1, F2, F5, F6, F11, F13	7
Fujii N [20]	2014	Japan	Cohort	13/465	F1, F5, F6, F7, F8, F10, F11, F12	8
Vieira AG [21]	2014	Brazil	Cohort	63/1,286	F1, F3, F4, F5, F7, F8, F9, F10, F12, F13	8
Rhee CK [22]	2016	Korea	Cohort	36/860	F1, F2, F3, F4, F5, F6, F7, F8, F9, F10	8
Bertelli L [23]	2017	Italy	Cohort	7/89	F1, F4, F7	8
Zhuang J [24]	2019	China	Cohort	25/444	F1, F2, F5, F6, F7, F8, F11, F12, F13	8
Pham J [25]	2021	Iran	Cohort	25/275	F1, F6, F10, F12	9
Huang QS [26]	2024	China	Case-control	195/780	F1, F3, F10, F12, F13	7

*Abbreviation*: *BOS* bronchiolitis obliterans syndrome, *NOS* Newcastle-Ottawa Scale

*Risk factors*: F1, female sex; F2, ABO blood group incompatibility; F3, unrelated donor; F4, human leukocyte antigen mismatched; F5, peripheral blood stem cell transplantation; F6, myeloablative conditioning; F7, total body irradiation; F8, busulfan; F9, methotrexate; F10, acute graft-versus-host disease; F11, grade Ⅱ-Ⅳ acute graft-versus-host disease; F12, chronic graft-versus-host-disease; F13, cytomegalovirus viremia

## Results of the meta-analysis

Comprehensive meta-analysis results for all examined variables are presented in Table 2, while forest plots depicting non-significant associations appear in the supplementary Material 2 (Figures S1–S6).

**Table 2:** Pooled analysis of each included risk factor for BOS

Risk factors	Number of included studies	Pooled effects			Heterogeneity		Analysis models
		OR	95% CI	*p* value	I^2^, %	*p* value	
Female sex	14	1.26	1.08, 1.47	0.003	19	0.25	Fixed-effect model
ABO blood group incompatibility	3	1.39	1.07, 1.81	0.01	0	0.95	Fixed-effect model
Unrelated donor	6	0.97	0.67, 1.41	0.87	40	0.14	Fixed-effect model
HLA mismatched donor	6	0.92	0.62, 1.39	0.70	42	0.12	Fixed-effect model
PBSCT	10	1.31	1.04, 1.64	0.02	26	0.21	Fixed-effect model
MAC	8	1.21	0.81, 1.81	0.35	52	0.04	Random-effect model
TBI	8	0.89	0.66, 1.21	0.47	0	0.52	Fixed-effect model
BU	7	1.25	0.94, 1.67	0.13	0	0.96	Fixed-effect model
MTX	3	1.34	0.72, 2.48	0.35	0	0.54	Fixed-effect model
aGVHD	7	1.93	1.16, 3.23	0.01	72	0.001	Random-effect model
Grade II-IV aGVHD	5	1.41	1.12, 1.77	0.004	0	0.70	Fixed-effect model
cGVHD	10	11.69	5.29, 25.82	< 0.00001	78	< 0.00001	Random-effect model
CMV viremia	6	0.88	0.72, 1.09	0.25	0	0.57	Fixed-effect model

*Abbreviation*: *aGVHD* acute graft-versus-host disease, *BU* busulfan, *CI* confidence interval, *cGVHD* chronic graft-versus-host-disease, *CMV* cytomegalovirus, *HLA* human leukocyte antigen, *MAC* myeloablative conditioning, *MTX* methotrexate, *OR* odd ratio, *PBSCT* peripheral blood stem cell transplantation, *TBI* total body irradiation

### Female sex

**Fig. 2:**
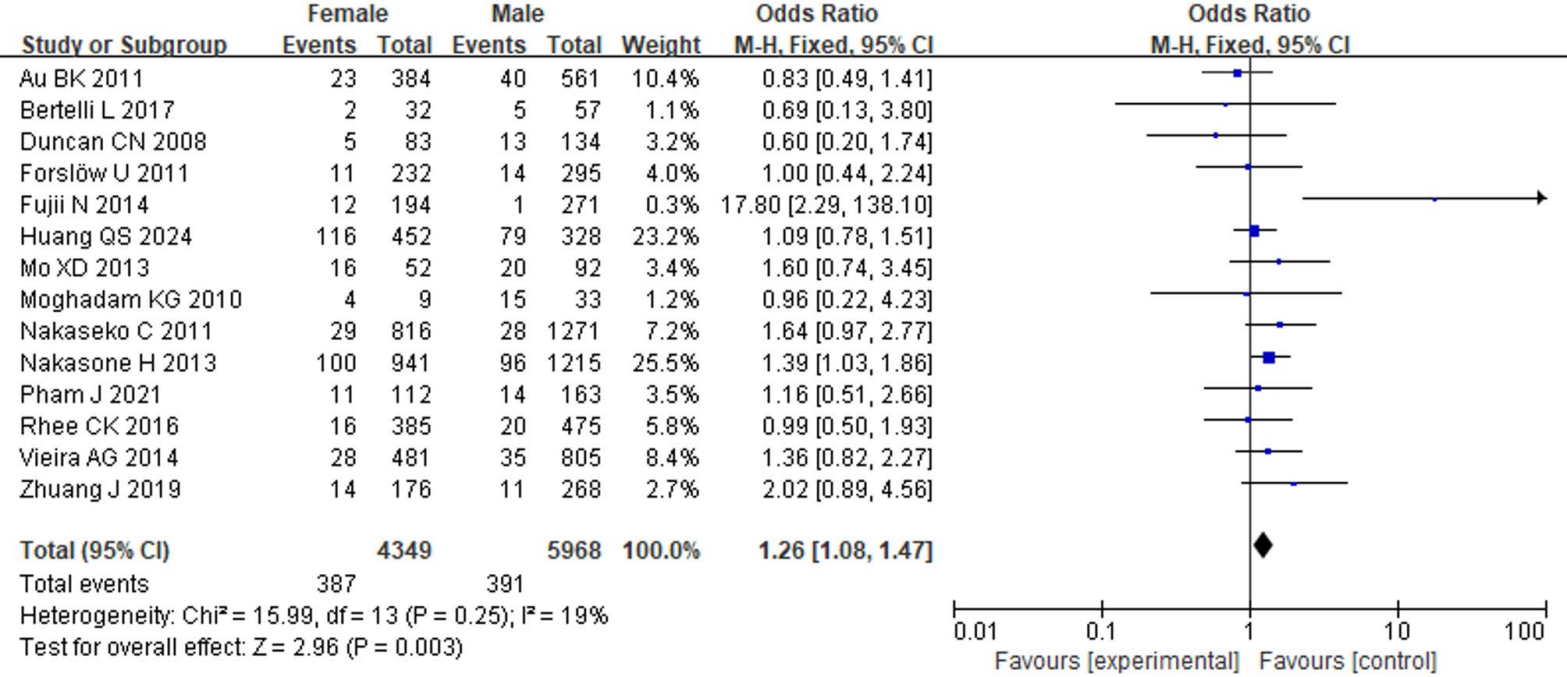
Forest Plot of female sex

Fourteen studies involving 10,317 patients were included. Given the low observed heterogeneity (*I*^2^ = 19%, *p* = 0.25), we used a fixed-effects model to pool the results. The results revealed that female patients had a higher risk of developing BOS compared to male patients (OR = 1.26; 95% CI: 1.08, 1.47; *p* = 0.003) (Fig. 2).

### ABO blood group incompatibility

**Fig. 3:**
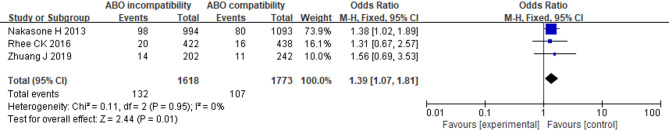
Forest Plot of ABO blood group incompatibility

Three studies with 3,391 patients were included. Given the absence of significant heterogeneity (*I*^2^ = 0%, *p* = 0.95), we pooled the results using a fixed-effects model. The analysis identified ABO blood group incompatibility in HSCT as significantly associated with increased BOS occurrence (OR = 1.39; 95% CI: 1.07, 1.81; *p* = 0.01) (Fig. 3).

### Unrelated donor

Six studies involving 3,701 patients were included. With moderate heterogeneity observed (*I*^2^ = 40%, *p* = 0.14), a fixed-effects model was used. The results revealed no significant difference in BOS occurrence between HSCT recipients with related versus unrelated donors (OR = 0.97; 95% CI: 0.67, 1.41; *p* = 0.87) (Figure S1).

### Human leukocyte antigen mismatched donor

Six studies with 2,737 patients were included. Given moderate heterogeneity across studies (*I*^2^ = 42%, *p* = 0.12), we used a fixed-effects model. Our findings revealed no significant correlation between human leukocyte antigen (HLA) mismatched donor and BOS occurrence (OR = 0.92; 95% CI: 0.62, 1.39; *p* = 0.70) (Figure S2).

### Peripheral blood stem cell transplantation

**Fig. 4:**
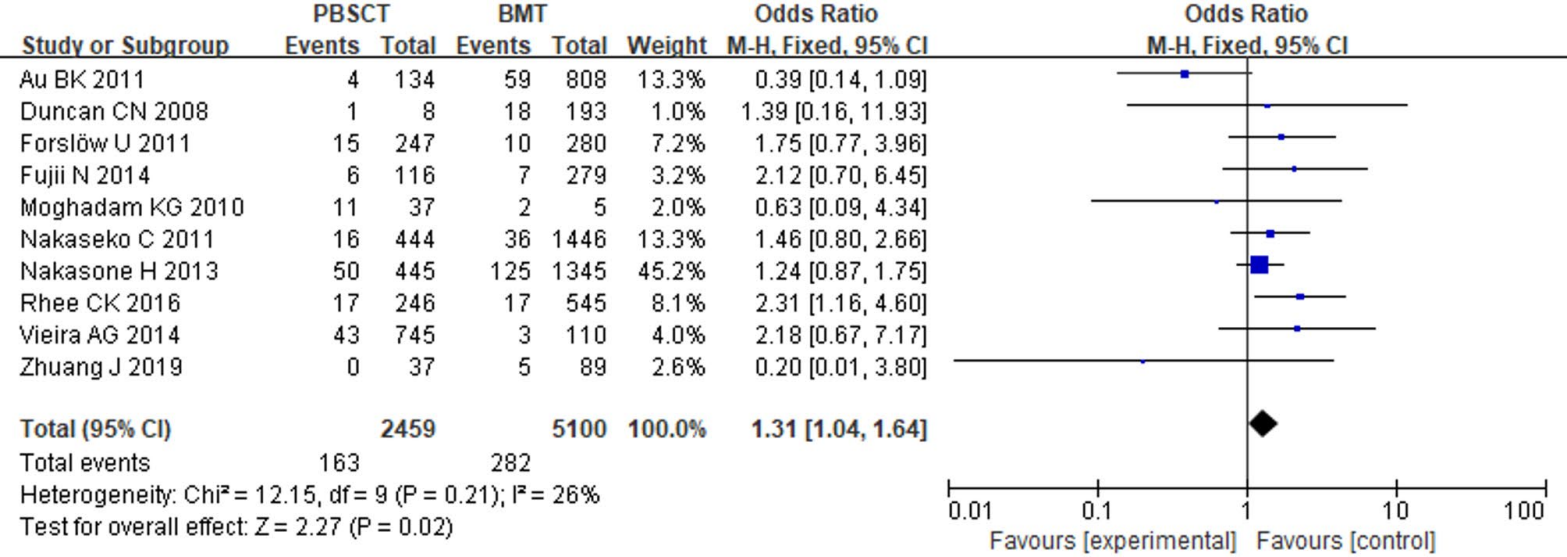
Forest Plot of PBSCT

Ten studies involving 7,559 patients were included. With minimal observed heterogeneity (*I*^2^ = 26%, *p* = 0.21), a fixed-effects model was used. The analysis demonstrated significantly greater BOS susceptibility among HSCT patients receiving peripheral blood stem cell transplantation (PBSCT) compared to bone marrow transplants (OR = 1.31; 95% CI: 1.04, 1.64; *p* = 0.02) (Fig. 4).

### Myeloablative conditioning

**Fig. 5:**
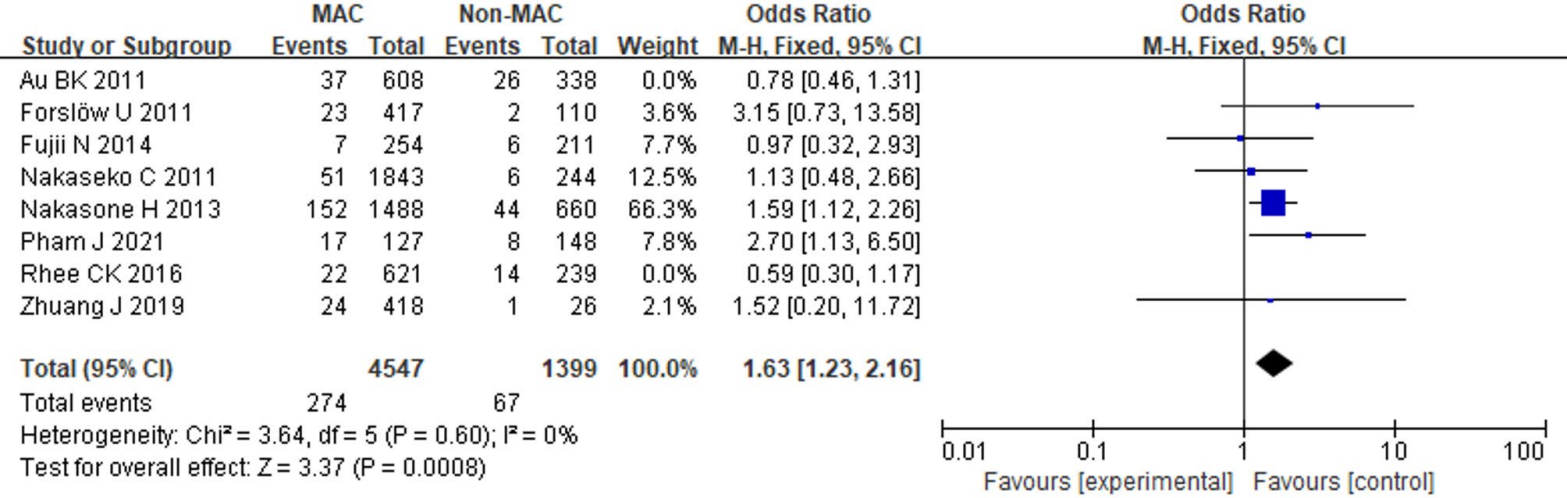
Forest Plot of MAC (After excluding the studies by Rhee et al. (2016) and Au et al. (2011))

Eight studies involving 7,752 patients were included. Due to high heterogeneity (*I*^2^ = 52%, *p* = 0.04), a random-effects model was used. The primary analysis showed no statistically significant association (OR = 1.21; 95% CI: 0.81, 1.81; *p* = 0.35). Subsequent sensitivity analysis demonstrated a significant reduction in heterogeneity after excluding the studies by Rhee et al. (2016) [22] and Au et al. (2011) [16]. Upon reanalysis excluding these two studies, myeloablative conditioning (MAC) was found to be associated with an increased risk of BOS (OR = 1.63; 95% CI: 1.23, 2.16; *p* = 0.0008; *I*^2^ = 0%, *p* = 0.60) (Fig. 5).

### Busulfan

Seven studies involving 5,834 patients were included. Analysis was performed using a fixed-effects model given the absence of significant heterogeneity (*I*^2^ = 0%, *p* = 0.96). The meta-analysis found no evidence linking busulfan (BU) administration to BOS occurrence (OR = 1.25; 95% CI: 0.94, 1.67; *p* = 0.13) (Figure S3).

### Total body irradiation

Eight studies involving 5,579 patients were included. Given the negligible heterogeneity (*I*^2^ = 0%, *p* = 0.52), a fixed-effects model was used. The results revealed no statistically significant relationship between total body irradiation (TBI) exposure and BOS development (OR = 0.89; 95% CI: 0.66, 1.21; *p* = 0.47) (Figure S4).

### Methotrexate

Three studies involving 2,284 patients were analyzed. A fixed-effects model was used since there was no significant heterogeneity (*I*^2^ = 0%, *P* = 0.54). The results revealed no statistically significant relationship between methotrexate (MTX) use and BOS development (OR = 1.34; 95% CI: 0.72, 2.48; *p* = 0.35) (Figure S5).

### aGVHD

**Fig. 6:**
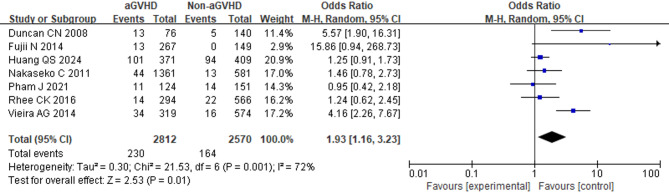
Forest Plot of aGVHD

**Fig. 7:**
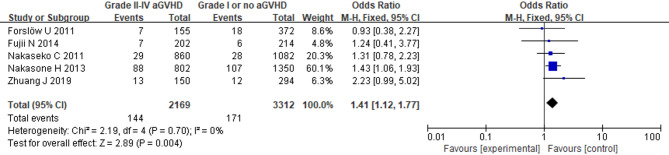
Forest Plot of grade II-IV aGVHD

Analysis of seven studies involving 5,382 patients demonstrated a significant correlation between aGVHD and BOS onset. (OR = 1.93; 95% CI: 1.16, 3.23; *p* = 0.01) (Fig. 6). Significant heterogeneity was observed in a random-effects model (*I*^2^ = 72%, *p* = 0.001), and sensitivity analysis indicated that overall heterogeneity remained substantial regardless of which study was removed. Additionally, five studies with 5,481 patients were analyzed to assess the association between grade II-IV aGVHD and BOS. The pooled results showed that patients with grade II-IV aGVHD had a higher risk of developing BOS compared to those with grade I or no aGVHD (OR = 1.41; 95% CI: 1.12, 1.77; *p* = 0.004) (Fig. 7). A fixed-effects model was used since no heterogeneity was observed (*I*^2^ = 0%, *p* = 0.70).

### Extrapulmonary chronic graft-versus-host disease

**Fig. 8:**
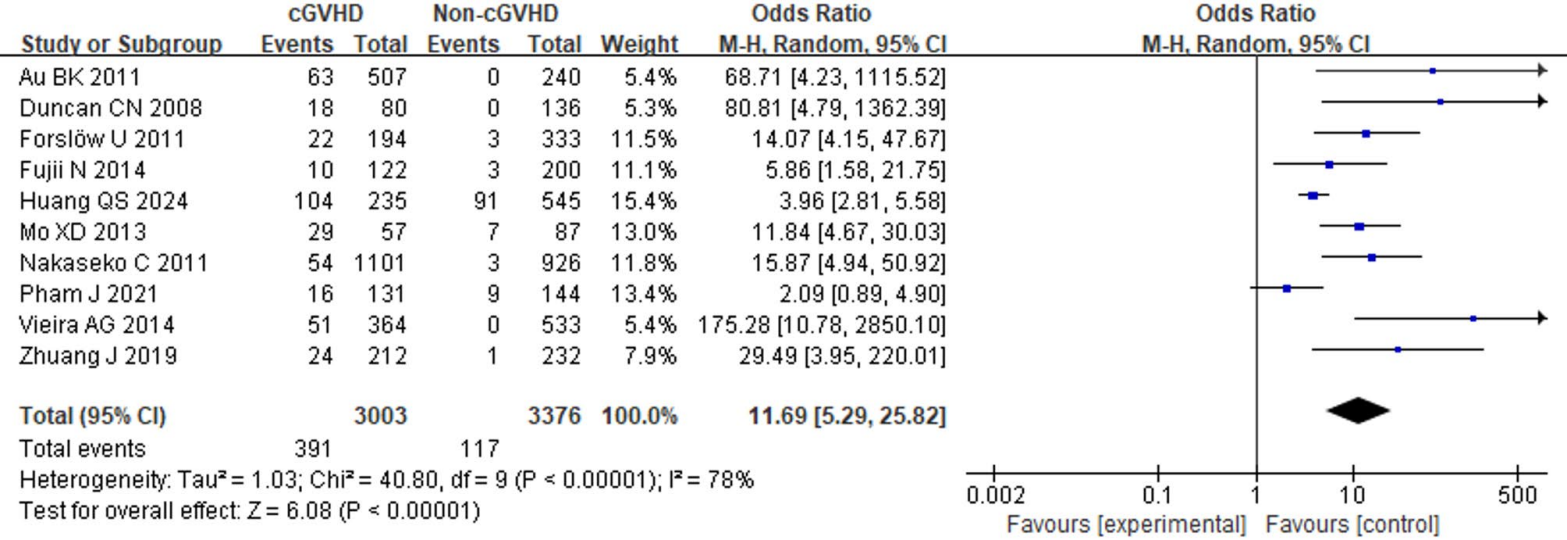
Forest Plot of extrapulmonary cGVHD

Ten studies involving 6,379 patients were included. However, substantial between-study heterogeneity was detected (*I*^2^ = 78%, *p* < 0.00001) and could not be reduced by excluding any single study. Consequently, we used a random-effects model to pool the results. The results demonstrated that patients with extrapulmonary chronic graft-versus-host disease (cGVHD) had a significantly increased incidence of BOS (OR = 11.69; 95% CI: 5.29, 25.82; *p* < 0.00001) (Fig. 8).

### Cytomegalovirus viremia

Six studies involving 4,779 patients were included. A fixed-effects model was used given the negligible heterogeneity (*I*^2^ = 0%, *p* = 0.57). The pooled results revealed no significant association between cytomegalovirus (CMV) viremia and BOS development (OR = 0.88; 95% CI: 0.72, 1.09; *p* = 0.25) (Figure S6).

### Heterogeneity testing and sensitivity analysis

**Fig. 9:**
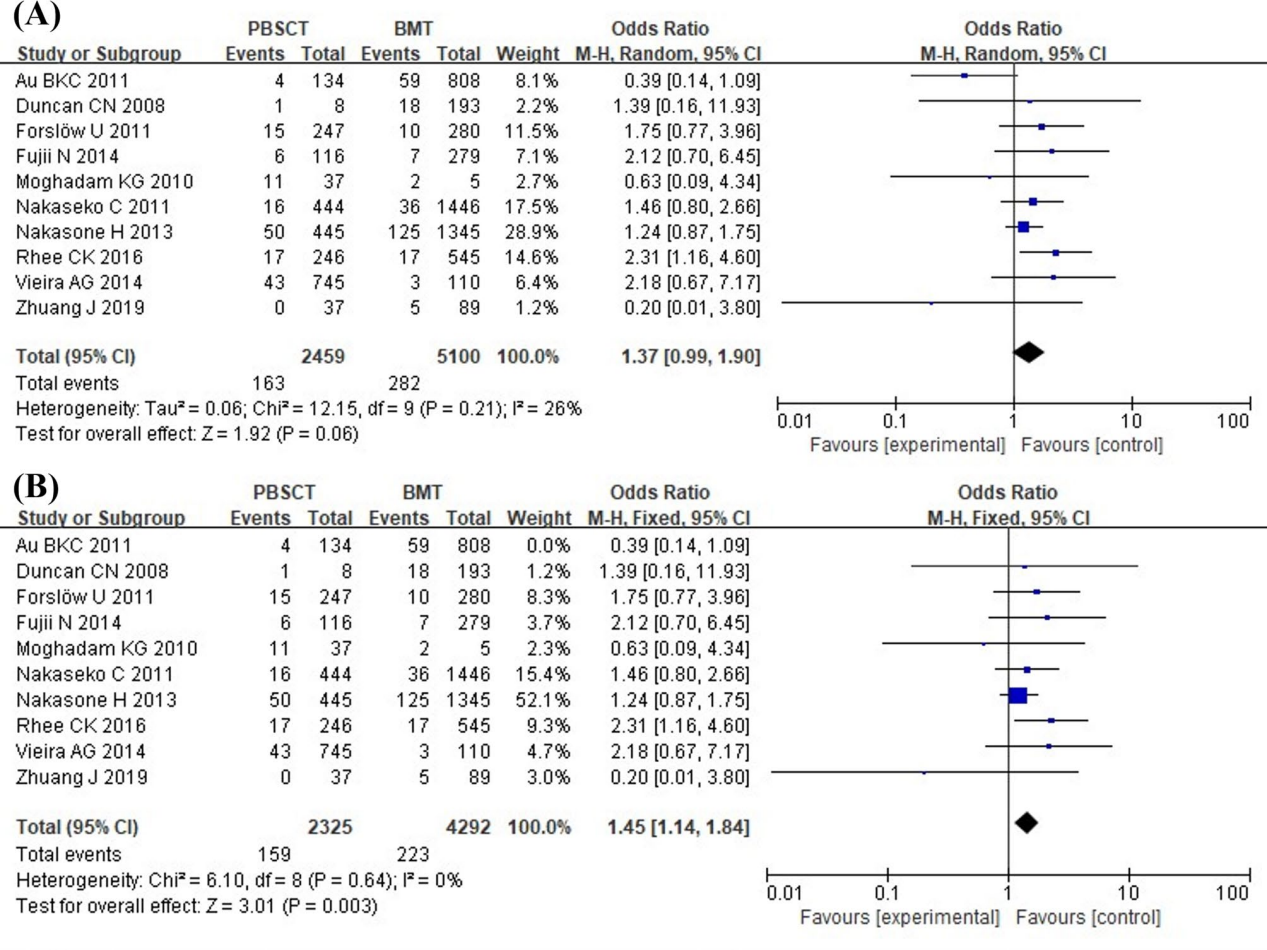
(**A**) Forest plot of PBSCT (Random-effects model), (**B**) Forest plot of PBSCT (After excluding the study by Au et al. (2011))

To assess the consistency of our results under different scenarios, we performed sensitivity analyses through two approaches. First, we performed a comparative analysis using both fixed- and random-effects modeling approaches, with detailed results presented in Table 3. Second, we systematically applied the leave-one-out method by sequentially excluding individual studies and recalculating the pooled estimates to assess their potential influence on the overall results [27]. Except for PBSCT, the direction and magnitude of combined estimates did not vary markedly with the use of different Model analyses and the removal of the studies, indicating that the meta-analyses had good validity and robustness. For PBSCT, the random-effects Model yielded a combined OR of 1.37 with a 95% CI of (0.99, 1.90) (Fig. 9 A), differing from the fixed-effects model results. Sensitivity analysis identified the study by Au et al. (2011) [16] as a potential source of heterogeneity. After reanalyzing the pooled results without this study, PBSCT was still associated with an increased risk of BOS (OR = 1.45; 95% CI: 1.14, 1.84; *p* = 0.003; *I*^2^ = 0%, *p* = 0.64) (Fig. 9B).

**Table 3:** Pooled results from Random-Effects and Fixed-Effects models

Risk factors	Random-Effects Models		Fixed-Effects Models	
	OR	95%CI	OR	95%CI
Female sex	1.23	1.02, 1.49	1.26	1.08, 1.47
ABO blood group incompatibility	1.39	1.07, 1.81	1.39	1.07, 1.81
Unrelated donor	0.99	0.57, 1.74	0.97	0.67, 1.41
HLA mismatched donor	1.00	0.55, 1.82	0.92	0.62, 1.39
PBSCT	1.37	0.99, 1.90	1.31	1.04, 1.64
MAC	1.61	1.21, 2.14	1.63	1.23, 2.16
TBI	0.87	0.64, 1.19	0.89	0.66, 1.21
BU	1.24	0.93, 1.66	1.25	0.94, 1.67
MTX	1.28	0.69, 2.37	1.34	0.72, 2.48
aGVHD	1.93	1.16, 3.23	1.67	1.34, 2.09
Grade II-IV aGVHD	1.41	1.12, 1.78	1.41	1.12, 1.77
cGVHD	11.69	5.29, 25.82	8.15	6.30, 10.55
CMV viremia	0.88	0.71, 1.09	0.88	0.72, 1.09

*Abbreviation*: *aGVHD *acute graft-versus-host disease, *BU* Busulfan, *CI* confidence interval, *cGVHD* chronic graft-versus-host-disease, *CMV* cytomegalovirus, *HLA* human leukocyte antigen, *MAC* myeloablative conditioning, *MTX* methotrexate, *OR* odd ratio, *PBSCT* peripheral blood stem cell transplantation, *TBI* total body irradiation

### Estimation of publication bias

**Fig. 10:**
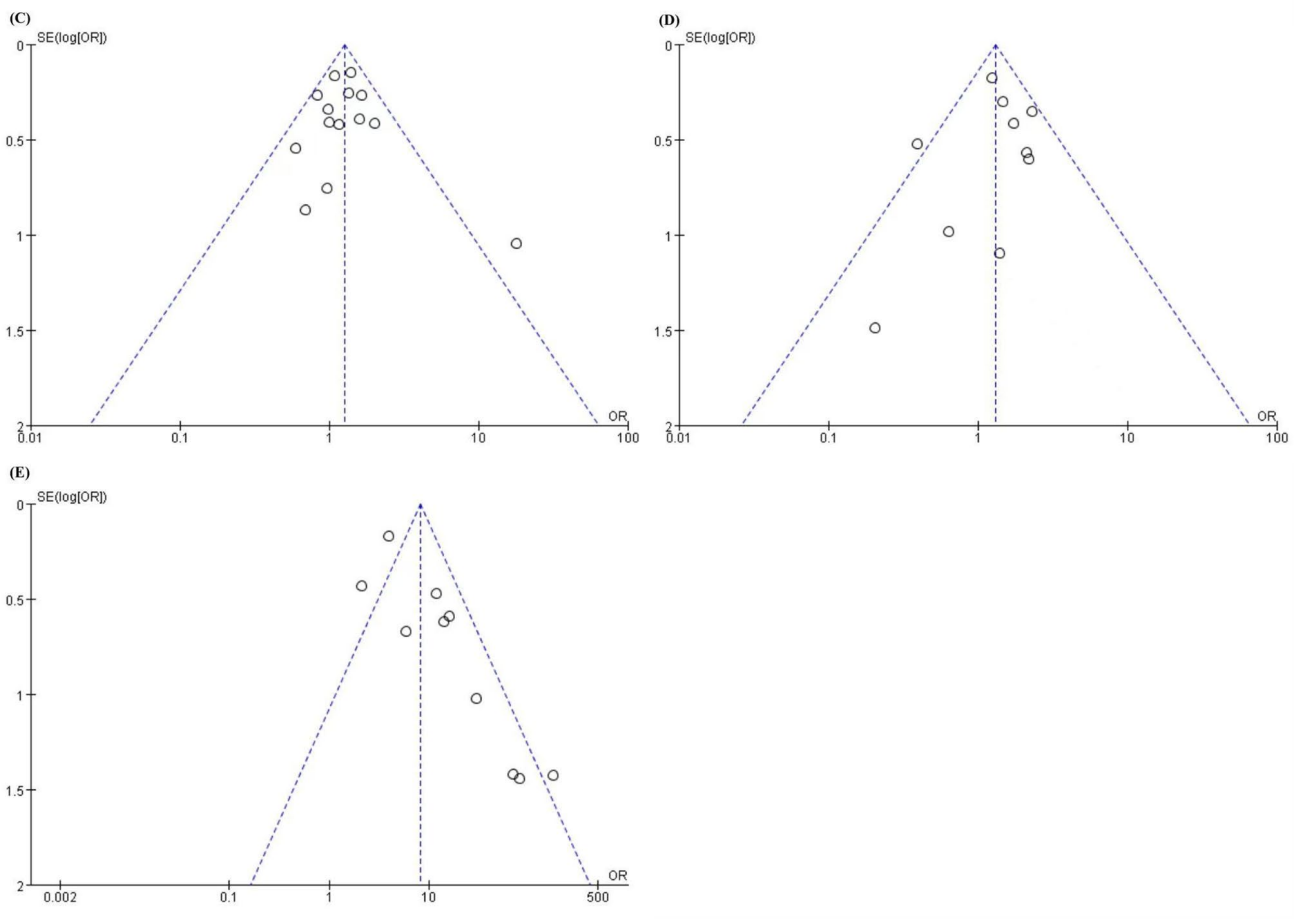
(**C**) Funnel Plots for Female sex, (**D**) Funnel Plots for PBSCT, (**E**) Funnel Plots for cGVHD

Publication bias was assessed using funnel plot visualization and Egger’s regression analysis for meta-analyses containing ≥ 10 studies. No significant bias was detected for female sex (Egger’s, *p* = 0.768; funnel plot symmetry) (Fig. 10 C). PBSCT analyses demonstrated minimal funnel plot asymmetry without statistical significance (Egger’s, *p* = 0.630) (Fig. 10D). Conversely, cGVHD analyses exhibited substantial funnel plot asymmetry (Fig. 10E), corroborated by statistically significant Egger’s test results (*p* = 0.004), indicating probable publication bias.

## Discussion

This meta-analysis systematically reviewed 14 studies comprising 10,317 HSCT patients. All included studies were of high quality, with NOS scores between 7 and 9. Our results identified the following seven variables as being associated with an increased risk of BOS: female sex, ABO blood group incompatibility, PBSCT, MAC, aGVHD, grade II-IV aGVHD, and extrapulmonary cGVHD.

### Female sex

Our analysis suggests that female patients are at higher risk of developing BOS compared to males. Although the exact mechanism remains unclear, potential factors may include sex hormones and differences in immune function [28]. Androgens, such as testosterone, may exert immunosuppressive effects, while estrogens tend to stimulate immune responses [29, 30]. This heightened immune activity in females could increase the risk of graft-versus-host disease (GVHD) and subsequently BOS. Additionally, genes located on the X chromosome and their epigenetic modifications may influence immune regulation and inflammation [31], potentially contributing to BOS development.

### ABO blood group incompatibility

ABO incompatibility is known to correlate with graft rejection in solid organ transplants [19, 32]. Our findings suggest a similar association with BOS following HSCT. In minor ABO-incompatible HSCT, donor-derived B cells may produce anti-recipient A/B antibodies that bind directly to pulmonary epithelial cells, initiating local immune injury [19, 33, 34]. In major ABO-incompatible transplantation, an alternative mechanism may involve inflammatory processes and the activation of adhesion molecules, initiated by the destruction of donor-derived erythrocytes and the formation of immune complexes with allo- or auto-reactive antibodies generated by residual recipient B cells [19, 35]. These inflammatory states, commonly observed in intravascular hemolysis, could contribute to thrombosis and platelet activation, ultimately inducing lung injury [19].

### PBSCT

In recent decades, bone marrow has been the primary source for HSCT, but the proportion of umbilical cord blood and peripheral blood has been gradually increasing [36]. Some reviews have indicated that PBSCT increases the incidence of BOS [37, 38], which is consistent with our pooled results. Compared with bone marrow, peripheral blood contains a higher proportion of cytotoxic CD8 + T cells, which may facilitate early immune recovery and potentially damage lung tissue [4, 39]. Additionally, granulocyte colony-stimulating factor administration during mobilization may promote Th2 cell differentiation. The resulting cytokines (e.g., IL-4, IL-13) could contribute to fibrotic processes that may be involved in BOS pathogenesis [40, 41].

### GVHD

GVHD, a key immune-mediated complication of HSCT, occurs when donor immune cells attack recipient tissues [42]. Both acute and chronic GVHD have been linked to BOS risk [6, 24, 43]. In aGVHD, direct immune-mediated injury to pulmonary tissue may lead to airway epithelial damage, followed by repair processes that, if excessive, can result in fibrosis [43–45]. aGVHD frequently evolves into cGVHD, during which persistent inflammatory injury and abnormal repair mechanisms may also target the lungs [46, 47]. Immunosuppressive agents used for GVHD management might additionally influence pulmonary immune status. Our analysis indicated that both aGVHD and cGVHD, particularly grade II–IV aGVHD, were associated with an increased risk of BOS. However, significant heterogeneity was observed among the included studies, which may affect the reliability of our findings. Despite our attempts to conduct sensitivity analyses and even subgroup meta-analyses, high heterogeneity still persists. Across the included studies, the diagnostic criteria for cGVHD were often poorly defined, limiting comparability. Additionally, significant publication bias was detected, which may have compromised the validity of our pooled estimates.

### MAC

MAC is an important step prior to HSCT. This procedure ablates existing marrow stem cells to facilitate donor cell engraftment and subsequent hematopoietic recovery in transplant recipients. Our findings indicated that MAC is associated with an increased risk of BOS. The mechanism might be associated with the direct lung injury caused by the high-dose chemotherapeutic agents and/or radiation therapy utilized in the conditioning regimen [4, 48]. MAC regimens often consist of alkylating agents and/or TBI [49]. As a commonly used alkylating agent in myeloablative conditioning regimens, BU can directly damage airway epithelial cells. Previous reviews have reported that BU-based conditioning regimens increase the risk of developing BOS by approximately 2.73 times [50]. Meanwhile, during TBI, the radiation directly causes lung injury, which can impair the immune repair function of the lungs, leading to BOS [48]. Although our meta-analysis results do not directly elucidate the relationship between BU, TBI, and BOS, which may be related to the dosage differences of BU and TBI in various studies, we can speculate that, since MAC regimens often consist of BU and/or TBI, there may be a certain association between them, and the use of BU or TBI in conditioning may increase the risk of developing BOS. However, additional studies are required to investigate the association between them.

Our analysis did not identify statistically significant associations between BOS and unrelated donors, HLA mismatch, MTX use, or CMV viremia. These findings may reflect variability in transplant protocols or limitations inherent in retrospective study designs. Overall, BOS development appears to involve a complex interplay of multiple factors, and the predictive value of individual risk factors remains limited. Further prospective, multicenter studies employing standardized diagnostic criteria are needed to validate and refine these associations.

## Limitation

This study has several limitations. First, the investigation of risk factors was constrained by the limited sample size, precluding analysis of less commonly studied or highly heterogeneous factors such as age, donor-recipient sex mismatch, and post-transplant pulmonary infections, which may affect the comprehensiveness of our findings. Second, all included studies were retrospective in design, which may introduce recall and selection biases. Third, although sensitivity analyses were conducted, substantial heterogeneity persisted, potentially limiting the reliability of our conclusions. Furthermore, the included studies were conducted across different decades, during which there have been significant advances in transplantation techniques, diagnostic methods, and supportive care. These temporal variations may introduce time bias, affecting the consistency and comparability of the included studies. Future studies should consider this factor when designing analyses and interpreting results. Lastly, due to the lack of access to individual patient-level data, we were unable to evaluate the combined effects of multiple risk factors. Understanding potential interactions or cumulative impacts of these variables will require individual participant data meta-analyses or large-scale prospective studies.

## Conclusions

Female sex, ABO blood group incompatibility, PBSCT, MAC, aGVHD (especially grade II–IV), and extrapulmonary cGVHD are associated with an increased risk of BOS after HSCT. The relationship between other potential risk factors and BOS remains to be clarified in future research.

## Supplementary Information

Supplementary Material 1.

Supplementary Material 2.

## Data Availability

All data generated or analyzed during this study are included in the published article and its supplementary material.

## Abbreviations

aGVHD: Acute graft-versus-host disease
BU: Busulfan
BO: Bronchiolitis obliterans
BOS: Bronchiolitis obliterans syndrome
CI: Confidence interval
CMV: Cytomegalovirus
cGVHD: Chronic graft-versus-host-disease
GVHD: Graft-versus-host-disease
HLA: Human leukocyte antigen
MTX: Methotrexate
MAC: Myeloablative conditioning
NIH: National Institutes of Health
OR: Odd ratio
PBSCT: Peripheral blood stem cell transplantation
PROSPERO: Database of Prospectively Registered Systematic Reviews
PRISMA: Preferred Reporting Items for Systematic Reviews and Meta-Analyses
TBI: Total body irradiation
