# Supplementary material for: Risk factors for bronchiolitis obliterans syndrome after hematopoietic stem cell transplantation: a systematic review and meta-analysis
Source: BMC Pulm Med. 2025 Oct 2;25:445. doi: 10.1186/s12890-025-03925-1 (PMC12492564; doi:10.1186/s12890-025-03925-1)
Supplement: Supplementary file 2 — Supplementary Material 2. [file 12890_2025_3925_MOESM2_ESM.docx]

**Supplementary material**

**Figure. S1-S6:** Forest plots of non-significant risk factors.

**Table S1:** Quality scores of included retrospective cohort studies using Newcastle-Ottawa Scale.

**Table S2:** Quality scores of included case-control studies using Newcastle-Ottawa Scale.

**Search strategies in PubMed:** (("Hematopoietic Stem Cell Transplantation"[Mesh]) OR (((((((hematopoietic stem cell transplantation[Title/Abstract]) OR (hemopoietic stem cell transplantation[Title/Abstract])) OR (bone marrow transplantation[Title/Abstract])) OR (peripheral blood hematopoietic stem cell transplantation[Title/Abstract])) OR (Umbilical cord blood stem cell transplantation[Title/Abstract])) OR (HSCT[Title/Abstract])) OR (hematopoietic SCT[Title/Abstract]))) AND (("Bronchiolitis Obliterans"[Mesh] OR "Bronchiolitis Obliterans Syndrome"[Mesh]) OR (((((((((((((Bronchiolitis Obliterans[Title/Abstract]) OR (Constrictive Bronchiolitis[Title/Abstract])) OR (Bronchiolitides, Constrictive[Title/Abstract])) OR (Bronchiolitis, Constrictive[Title/Abstract])) OR (Constrictive Bronchiolitides[Title/Abstract])) OR (Bronchiolitis, Exudative[Title/Abstract])) OR (Bronchiolitides, Exudative[Title/Abstract])) OR (Exudative Bronchiolitides[Title/Abstract])) OR (Exudative Bronchiolitis[Title/Abstract])) OR (Bronchiolitis, Proliferative[Title/Abstract])) OR (Bronchiolitides, Proliferative[Title/Abstract])) OR (Proliferative Bronchiolitides[Title/Abstract])) OR (Proliferative Bronchiolitis[Title/Abstract])))


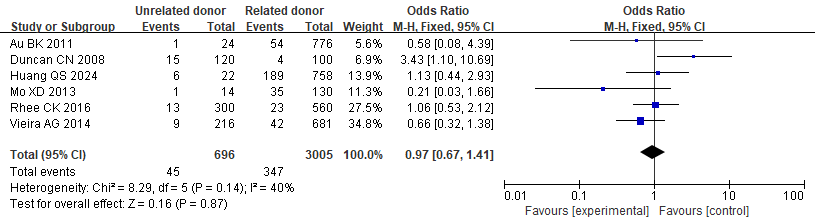


**Figure. S1.** Forest plot of unrelated donor.

**
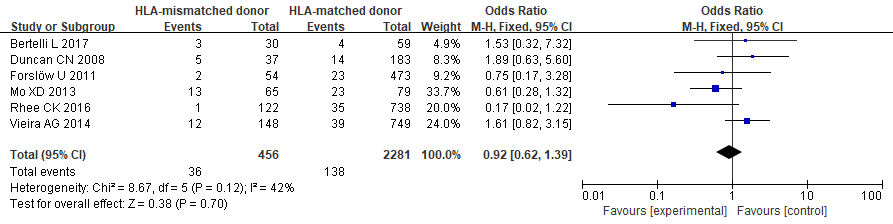
**

**Figure. S2.** Forest plot of human leukocyte antigen (HLA) mismatched donor.


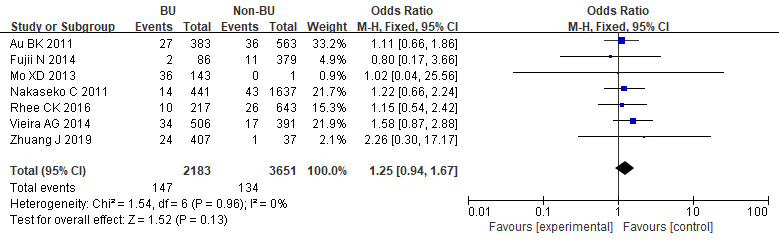


**Figure. S3.** Forest plot of busulfan (BU).


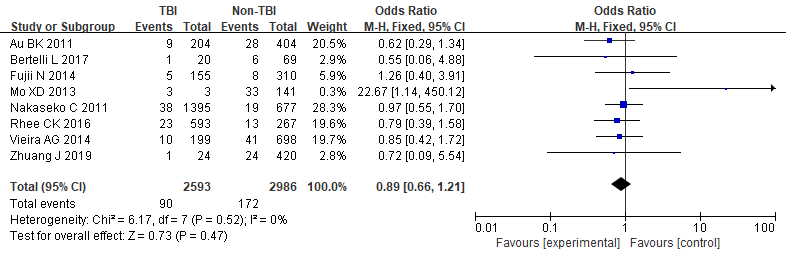


**Figure. S4.** Forest plot of total body irradiation (TBI).

**
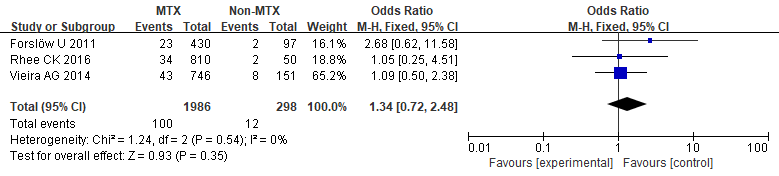
**

**Figure. S5.** Forest plot of methotrexate (MTX).


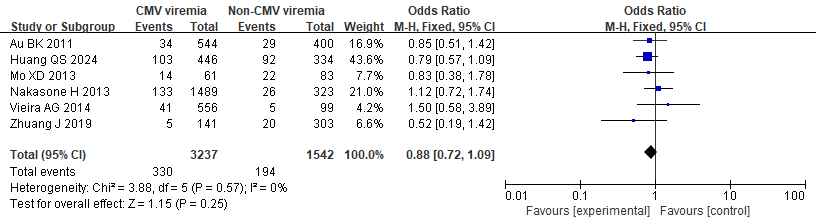


**Figure. S6.** Forest plot of cytomegalovirus (CMV) viremia.

**Table S1**. Quality scores of included retrospective cohort studies using Newcastle-Ottawa Scale.

| Study | Selection | | | | Comparability | | Outcome | | NOS |
| --- | --- | --- | --- | --- | --- | --- | --- | --- | --- |
|  | Representativeness of the exposed cohort | Selection of the non- exposed cohort | Ascertainment of exposure | Demonstration that outcomes was not present at start of study | Comparability of cohorts on the basis of the design or analysis | Assessment of outcome | Adequate follow-up duration | Adequate of follow up of cohorts | overall scores |
| Duncan CN | 1 | 1 | 1 | 1 | 0 | 1 | 1 | 1 | 7 |
| Moghadam KG | 1 | 1 | 1 | 1 | 0 | 1 | 1 | 1 | 7 |
| Forslöw U | 1 | 1 | 1 | 1 | 1 | 1 | 1 | 1 | 8 |
| Au BKC | 1 | 1 | 1 | 1 | 1 | 1 | 1 | 1 | 8 |
| Nakaseko C | 1 | 1 | 1 | 1 | 1 | 1 | 1 | 1 | 8 |
| Fujii N | 1 | 1 | 1 | 1 | 1 | 1 | 1 | 1 | 8 |
| Vieira AG | 1 | 1 | 1 | 1 | 1 | 1 | 1 | 1 | 8 |
| Rhee CK | 1 | 1 | 1 | 1 | 1 | 1 | 1 | 1 | 8 |
| Bertelli L | 1 | 1 | 1 | 1 | 1 | 1 | 1 | 1 | 8 |

**Table. S1.** **(continued)**

| Study | Selection | | | | Comparability | | Outcome | | NOS |
| --- | --- | --- | --- | --- | --- | --- | --- | --- | --- |
|  | Representativeness of the exposed cohort | Selection of the non- exposed cohort | Ascertainment of exposure | Demonstration that outcomes was not present at start of study | Comparability of cohorts on the basis of the design or analysis | Assessment of outcome | Adequate follow-up duration | Adequate of follow up of cohorts | overall scores |
| Zhang J | 1 | 1 | 1 | 1 | 1 | 1 | 1 | 1 | 8 |
| Pham J | 1 | 1 | 1 | 1 | 2 | 1 | 1 | 1 | 9 |

Abbreviations: NOS: Newcastle-Ottawa Scale

**Table. S2.** Quality scores of included case-control studies using Newcastle-Ottawa Scale.

| Study | Selection | | | | Comparability | | Exposure | | NOS |
| --- | --- | --- | --- | --- | --- | --- | --- | --- | --- |
|  | Is the case definition adequate? | Representativeness of the cases | Selection of Controls | Definition of Controls | Comparability of cases and controls on the basis of the design or analysis | Ascertainment of exposure | Same method of ascertainment for cases and controls | Non-Response rate | overall scores |
| Nakasone H | 1 | 1 | 0 | 1 | 1 | 1 | 1 | 1 | 7 |
| Mo XD | 1 | 1 | 0 | 1 | 1 | 1 | 1 | 1 | 7 |
| Huang QS | 1 | 1 | 0 | 1 | 1 | 1 | 1 | 1 | 7 |

Abbreviations: NOS: Newcastle-Ottawa Scale
